# Mild Encephalitis/Encephalopathy With a Reversible Splenial Lesion of the Corpus Callosum (MERS): A Report of Two Pediatric Cases

**DOI:** 10.7759/cureus.104951

**Published:** 2026-03-09

**Authors:** Amine Kaake

**Affiliations:** 1 Pediatric Neurology, Hôpital Robert-Debré, AP-HP, Paris, FRA

**Keywords:** mers, mild encephalopathy/aseptic encephalitis with reversible splenial lesion of the corpus callosum, neurology and pediatric neurology, reversible splenial lesion syndrome, viral encephalopathy

## Abstract

Mild encephalitis/encephalopathy with a reversible splenial lesion of the corpus callosum (MERS) is an uncommon clinico-radiological syndrome marked by transient lesions of the splenium, which are typically demonstrated as diffusion-restricted hyperintensities on MRI. It presents with acute neurological symptoms such as seizures, dysarthria, or ataxia, often following viral infections. We report two pediatric cases of MERS observed in the ED. The first was a 23-month-old boy with focal status epilepticus in the setting of acute gastroenteritis. The second was an eight-year-old boy who presented with dysarthria, gait ataxia, and sensorimotor deficits after a febrile viral illness. Brain MRI in both patients showed diffusion-restricted lesions in the splenium of the corpus callosum, with no contrast enhancement. CSF and infectious workups were unremarkable, except for positive respiratory viral PCR in one case. Neurological recovery was rapid and complete in both children. Follow-up MRIs demonstrated full resolution of the lesions. These clinical and radiological patterns confirmed the diagnosis of MERS. These cases highlight the importance of recognizing MERS as a reversible and self-limiting cause of pediatric encephalopathy. Early MRI is crucial for diagnosis, avoiding unnecessary interventions, and guiding supportive management. Awareness of MERS and its typical imaging features is essential for clinicians and radiologists dealing with pediatric neurological presentations.

## Introduction

Mild encephalitis/encephalopathy with a reversible splenial lesion of the corpus callosum (MERS) is a clinico-radiological syndrome characterized by transient, focal lesions of the splenium of the corpus callosum, typically seen on MRI [1]. These lesions usually present as diffusion-restricted hyperintensities on diffusion-weighted imaging (DWI) with reduced apparent diffusion coefficient (ADC) values. Clinically, MERS is associated with acute neurological symptoms such as seizures, confusion, dysarthria, or ataxia, often following prodromal gastrointestinal or respiratory symptoms of viral origin. Although MERS is increasingly recognized, it remains relatively uncommon, particularly in the pediatric population. The true incidence is not well established, but most cases have been reported in East Asian pediatric cohorts, with sporadic reports worldwide.

MERS is considered part of the broader spectrum of reversible splenial lesion syndrome, which encompasses various conditions associated with transient splenial abnormalities on MRI [2]. However, MERS specifically refers to cases associated with mild encephalopathy and favorable clinical outcomes. Reporting additional pediatric cases remains important to improve recognition of this condition, particularly in emergency settings where its presentation may mimic more serious neurological disorders such as stroke or encephalitis.

A large pediatric cohort study by Tada et al. demonstrated that MERS in children often presents with benign clinical evolution and complete radiological resolution, provided that appropriate supportive management is initiated early [1]. In addition, Garcia-Monco et al. emphasized the diagnostic utility of high b-value DWI in better delineating splenial lesions and monitoring their progression, reinforcing MRI as the imaging modality of choice for both diagnosis and follow-up [2]. While many cases of MERS have been described in adults, pediatric cases remain relatively uncommon in the literature. Here, we present two pediatric cases of MERS, highlighting the diverse clinical presentations and favorable outcomes associated with this condition. The first patient was a 23-month-old boy who presented with focal status epilepticus with secondary bilateralization, associated with symptoms of acute gastroenteritis lasting for two days. The second patient was an eight-year-old child who presented with acute neurological deficits, including dysarthria and ataxia, in a febrile context that had been evolving for 48 hours.

## Case presentation

Case 1

The first patient was a 23-month-old boy, born at 39 weeks of gestation, with no neonatal, medical, or family history. Growth parameters were within normal limits, and psychomotor development was age-appropriate. He presented to the pediatric ED in March 2021 with two generalized seizures during the night, each lasting less than five minutes, without associated fever, in the context of acute gastroenteritis that had been ongoing for 48 hours. Upon arrival, he was afebrile, with normal vital signs and blood glucose. Physical examination was unremarkable. On neurological examination, the patient was alert with a normal level of consciousness. Cranial nerve examination was normal. Muscle tone and strength were appropriate for age, with no focal motor deficit. Deep tendon reflexes were symmetric and normal. No abnormal movements were observed, and coordination appeared appropriate for age. No signs of meningeal irritation were present. An ECG was performed and found to be normal. During hospitalization for observation, the patient experienced recurrent seizures, described by the medical team as generalized tonic-clonic seizures without recovery of baseline consciousness between episodes.

**Figure 1:**
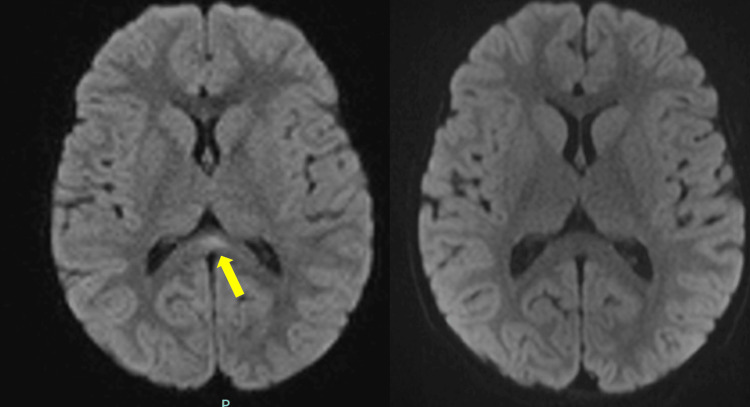
Case 1: Axial DWI of the brain (A) Initial MRI (March 2021): focal diffusion-restricted hyperintensity involving the splenium of the corpus callosum. (B) Follow-up MRI (June 2021): complete resolution of the previously described splenial diffusion restriction. DWI, diffusion-weighted imaging

This clinical presentation was consistent with status epilepticus, prompting initiation of continuous intravenous clonazepam infusion to achieve seizure control. A comprehensive etiological workup was performed. Blood tests showed normal electrolyte levels, renal function, calcium-phosphate-magnesium balance, and inflammatory markers (CRP negative). Blood lactate and pyruvate levels were within the physiological range. Amino acid chromatography was unremarkable, and urinary organic acid analysis revealed only ketosis. A CBC was normal, and urinary toxicology screening was negative. CSF analysis was normal: clear appearance, negative direct examination, sterile at 48 hours, mildly elevated glucose (4.8 mmol/L), no leukocytes, and 4 red blood cells/mm³. Multiplex PCR for HSV-1, HSV-2, VZV, CMV, and EBV was negative. A broad infectious workup revealed the presence of respiratory syncytial virus and a seasonal non-COVID-19 coronavirus on RT-PCR from a nasal swab. Stool virology was negative. Brain MRI revealed a focal DWI hyperintensity with restricted diffusion isolated to the splenium of the corpus callosum, with no other abnormalities (Figure 1A). Clinical evolution was favorable, with no seizure recurrence. A follow-up brain MRI performed three months later was normal, showing complete resolution of the splenial lesion (Figure 1B). Based on the clinical and radiological findings, a diagnosis of MERS (mild encephalitis with a reversible splenial lesion) was established.

Case 2

**Figure 2:**
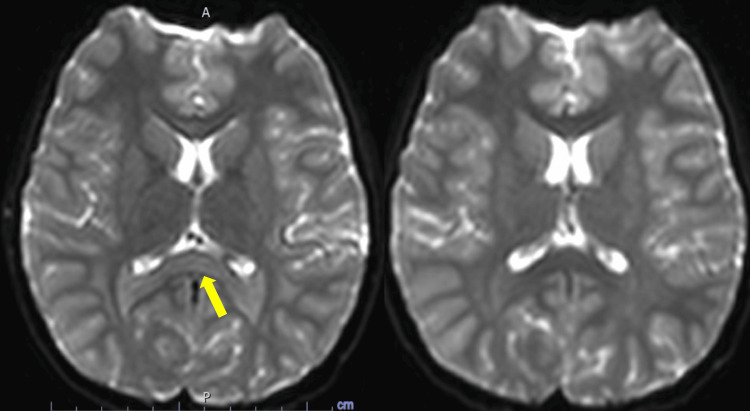
Case 2: Axial DWI of the brain (A) Initial MRI (September 2019): diffusion-restricted hyperintensity involving the splenium of the corpus callosum. (B) Follow-up MRI (October 2019): complete resolution of the diffusion restriction in the splenium of the corpus callosum. DWI, diffusion-weighted imaging

The second patient was an eight-year-old boy with a history of asthma treated with salbutamol as needed. His vaccinations were up to date. He had normal growth and psychomotor development, was in third grade (CE2), and had no personal or family history of neurological disorders. He presented to the pediatric ED in September 2019 with acute neurological symptoms. After returning home from school, he developed dysarthria, without word-finding difficulty or altered consciousness, followed shortly by ataxia with an unsteady gait and left upper limb motor weakness accompanied by hypoesthesia. He had been experiencing gastrointestinal viral symptoms for 48 hours, including low-grade fever, vomiting, and abdominal pain. His parents called emergency services, and a stroke alert was triggered. An urgent brain MRI was performed approximately two hours after symptom onset, which ruled out stroke but revealed hyperintensity on DWI with restricted diffusion involving the splenium of the corpus callosum and the periventricular subcortical white matter (centrum semiovale), without pathological contrast enhancement (Figure 2A).

Upon arrival at the pediatric ED following the MRI, the patient was alert and exhibited normal consciousness. A biological workup was performed, showing normal blood electrolyte levels and a normal CBC (hemoglobin at 12.6 g/dL), along with a mild inflammatory response (CRP at 12.9 mg/L, negative procalcitonin). CSF analysis was normal: clear appearance, negative direct examination, sterile culture at 48 hours, normal glycorrhachia, 1 white blood cell/mm³, and 0 red blood cells/mm³. Multiplex PCR for HSV-1, HSV-2, VZV, CMV, and EBV was negative. As part of the etiological investigation, blood serologies for EBV, CMV, VZV, *Chlamydia pneumoniae*, *Mycoplasma pneumoniae*, and Lyme disease were all negative. A pediatric neurology consultation was performed 35 hours after symptom onset and found a normal neurological examination, except for mild dysmetria on finger-to-nose testing on the left side and slight gait ataxia. Clinical evolution was favorable. A follow-up brain MRI was performed on October 21, 2019, which showed complete resolution of the splenial diffusion-weighted hyperintensity, with no residual abnormalities on T1- or T2-weighted sequences. The diffusion restriction in the subcortical white matter of the centrum semiovale had resolved, and the FLAIR hyperintensities had decreased in size and intensity (Figure 2B). This second case confirmed the diagnosis of MERS, with transient MRI lesions of the splenium of the corpus callosum and neurological symptoms in the context of a viral infection.

## Discussion

MERS is increasingly recognized as a distinct clinico-radiological syndrome. The two cases presented here illustrate the typical features of pediatric MERS: acute onset of neurological symptoms in the context of viral infection, radiological evidence of reversible splenial diffusion restriction, and complete clinical recovery without sequelae. Although the precise pathophysiology remains incompletely understood, several mechanisms have been proposed. Transient intramyelinic edema due to a cytokine-mediated inflammatory response, metabolic disturbances such as hyponatremia, and excitotoxic injury have all been suggested as potential contributors [3,4]. Importantly, the absence of significant inflammatory changes in CSF, as seen in both of our patients, is consistent with previously reported pediatric series [1].

The radiological hallmark of MERS is a hyperintense lesion in the splenium of the corpus callosum on DWI, with corresponding low ADC values. In some cases, additional lesions may be observed in the centrum semiovale or other white matter tracts, as demonstrated in our second patient. This extension beyond the splenium, sometimes referred to as MERS type 2, has been associated with a slightly more severe clinical presentation, although prognosis generally remains favorable [5]. Differential diagnoses include ischemic stroke, demyelinating disease (such as acute disseminated encephalomyelitis), metabolic disorders, and infectious encephalitis. Rapid recognition of the characteristic MRI pattern is crucial to avoid unnecessary invasive investigations or aggressive therapies. Both of our cases highlight the importance of considering MERS in the acute evaluation of pediatric patients with seizures or focal neurological deficits.

Management of MERS is essentially supportive, focusing on seizure control, hydration, and monitoring of electrolytes. The benign and self-limited course observed in our patients aligns with most of the pediatric literature [1,6]. Nevertheless, follow-up imaging is recommended to confirm resolution of lesions and to exclude alternative diagnoses. The cases we report add to the growing body of evidence that MERS should be recognized as a reversible and favorable condition, even in children presenting with alarming acute neurological symptoms. Increasing awareness among emergency physicians, neurologists, and radiologists is essential to ensure timely diagnosis and appropriate management.

Both cases illustrate the typical clinical and radiological features of pediatric MERS but also highlight differences in presentation. The first case presented with focal status epilepticus in the context of gastroenteritis, while the second case presented with focal neurological deficits mimicking acute stroke. In both patients, MRI demonstrated reversible splenial diffusion restriction with complete radiological resolution and favorable clinical outcomes.

## Conclusions

We report two typical pediatric cases of MERS, characterized by acute neurological symptoms and transient, specific MRI lesions, most often occurring in the context of a viral infection, with a favorable clinical outcome. These cases exhibit the same features as those described in the adult neurology literature. MERS is a rare syndrome with a characteristic clinical and radiological presentation. A variety of pathogens may be implicated, but in all cases, the neurological course is generally benign. MRI is the imaging modality of choice for both initial diagnosis and follow-up. It is essential that radiologists and neurologists be familiar with the radiological features of MERS and its broad range of etiologies to avoid unnecessary treatments and investigations.
